# The Long-Term Effects of Prenatal Hypoxia on Coronary Artery Function of the Male and Female Offspring

**DOI:** 10.3390/biomedicines10123019

**Published:** 2022-11-23

**Authors:** Nataliia Hula, Ricky Liu, Floor Spaans, Mazhar Pasha, Anita Quon, Raven Kirschenman, Christy-Lynn M. Cooke, Sandra T. Davidge

**Affiliations:** 1 Department of Physiology, University of Alberta, Edmonton, AB T6G 2R3, Canada; 2 Department of Obstetrics and Gynecology, Women and Children’s Health Research Institute, University of Alberta, Edmonton, AB T6G 2R3, Canada

**Keywords:** pregnancy complications, prenatal hypoxia, cardiovascular dysfunction, developmental origins of health and disease (DOHaD), offspring, coronary artery function, endothelin-1 (ET-1), endothelial dysfunction, sex differences

## Abstract

Prenatal hypoxia predisposes the offspring to the development of cardiovascular (CV) dysfunction in adult life. Using a rat model, we assessed the effect of prenatal hypoxia on vasoconstrictive and vasodilative mechanisms in left anterior descending coronary arteries of 4- and 9.5-month-old offspring. Endothelium-dependent relaxation to methylcholine and vasoconstriction responses to endothelin-1 (ET-1) were assessed by wire myography. Prenatal hypoxia impaired endothelium-dependent vasodilation in 4- and 9.5-month-old offspring. Inhibition of nitric oxide (NO) synthase prevented coronary artery relaxation in all groups. Inhibition of prostaglandin H synthase (PGHS) improved relaxation in prenatally hypoxic males and tended to improve vasorelaxation in females, suggesting that impaired vasodilation was mediated via increased PGHS-dependent vasoconstriction. An enhanced contribution of endothelium-dependent hyperpolarization to coronary artery vasodilation was observed in prenatally hypoxic males and females. No changes in endothelial NO synthase (eNOS) and PGHS-1 expressions were observed, while PGHS-2 expression was decreased in only prenatally hypoxic males. At 4 months, ET-1 responses were similar between groups, while ET_B_ inhibition (with BQ788) tended to decrease ET-1-mediated responses in only prenatally hypoxic females. At 9.5 months, ET-1-mediated responses were decreased in only prenatally hypoxic females. Our data suggest that prenatal hypoxia has long-term similar effects on the mechanisms of impaired endothelium-dependent vasodilation in coronary arteries from adult male and female offspring; however, coronary artery contractile capacity is impaired only in prenatally hypoxic females. Understanding the mechanistic pathways involved in the programming of CV disease may allow for the development of therapeutic interventions.

## 1. Introduction

Pregnancy-related complications severely impact the health and lifespan of offspring [1]. Fetal hypoxia is a major consequence of complicated pregnancies that affects normal fetal development and can result in the restriction of fetal growth [2]. Previous research demonstrated that fetal hypoxia leads to changes in offspring cardiovascular (CV) function and predisposes the offspring to the development of CV dysfunction in later life [3,4,5,6,7,8,9]; however, the mechanisms remain to be fully elucidated. 

Seminal research by Barker et al. demonstrated a strong association between disproportional (human) growth at birth and predisposition to the development of ischemic heart disease (IHD) [10]. In particular, a low birth weight, short birth length, small head circumference at birth, and low ponderal index were associated with increased risk of IHD and coronary heart disease (CHD) in adulthood [10,11], while an increase in birth weight correlated with a proportional reduction in the rate of IHD and CHD [12,13]. In humans, complicated pregnancies have been associated with an enhanced coronary blood flow in the fetus and in neonates [14,15,16]. Moreover, previous research in various animal models of pregnancy complications reported left ventricular (LV) hypertrophy and an increased coronary artery vascular reactivity (in lambs [17] and fetal sheep [18]), decreased fetal LV capillarization (in fetal rabbit [19]) and total myocardial capillary length (in rat neonates [20]), increased passive coronary arterial stiffness (in lambs [21]), and substantial structural remodeling of coronary vascular tree (in fetal rabbit [22]). However, little is known regarding the long-term effects of complicated pregnancies on adult offspring coronary artery function. One study showed that prenatal cocaine exposure results in sex-dependent dysfunction of coronary autoregulation in adult offspring. For instance, prenatal cocaine exposure caused a decrease in coronary myogenic tone in male offspring, but resulted in increased coronary myogenic tone in the females [23]. Moreover, another study reported that prenatal hypoxia was associated with attenuated serotonin- and protein kinase C (PKC) agonist-mediated contractile responses in the coronary arteries of adult male offspring (female offspring were not assessed) [24]. Taking into account that coronary circulation is essential in maintaining proper cardiac performance and represents ~5% of total cardiac output [25], the dysfunction of coronary arteries has been attributed to the development of myocardial ischemia [26] and severe clinical outcomes.

The vascular endothelium plays a major role in maintaining vascular function and homeostasis. Primarily, the vascular endothelium is involved in the production and release of endothelium-derived relaxing factors (such as nitric oxide (NO), prostacyclin- PGI_2_, and endothelium-derived hyperpolarization (EDH)), and endothelium-derived constricting factors (such as endothelin-1 (ET-1) and thromboxane A_2_)) [27]. An imbalance between vasodilation and vasoconstriction has been implicated in the development of endothelial dysfunction [28], resulting in CV dysfunction [29]. Previous reports suggest that offspring exposed to prenatal hypoxia are more prone to the development of hypertension [30], accompanied by an enhanced vasoconstrictor reactivity to big ET-1 (a precursor of ET-1) and an impaired NO-dependent endothelial function in the systemic circulation [4,30]. However, the link between prenatal hypoxia and coronary artery function in adult male and female offspring has not been fully explored. In the current study, we hypothesized that prenatal hypoxia impairs coronary artery function in adult offspring by reducing endothelium-dependent vasodilation and constrictor capacity in adult (4- and 9.5-month-old) male and female offspring. 

## 2. Materials and Methods

### 2.1. Animal Model of Prenatal Hypoxia

Three-month-old female Sprague–Dawley rats (Charles River, Kingston, NY; Raleigh, NC; Hollister, CA, USA) were mated overnight by housing with 3-month-old Sprague–Dawley males. Pregnancy was confirmed the following morning by the presence of sperm in the vaginal smear (designated as gestational day [GD] 0). Throughout the pregnancy period, dams were single housed in standard rat cages under a 10:14 h light–dark cycle and fed standard rat chow ad libitum. Pregnant rats were randomly divided into two groups: normoxic controls (housed at atmospheric oxygen throughout pregnancy: 21% O_2_; normoxia group) or hypoxic dams (exposed to hypoxia from GD 15–21 by placing them in a hypoxic chamber: 11% O_2_; p-hypoxia group). The pregnant rats were removed from the hypoxic chamber on GD 21 (term = GD 22) and were allowed to give birth in standard housing and oxygen conditions. At birth, litter size was decreased to 8 pups/litter (4 males and 4 females) to standardize postnatal conditions. On postnatal day 21, offspring were weaned, separated by sex, and double housed in standard housing conditions until either 1) ~4 months or 2) ~9.5 months of age. At 4 or 9.5 months of age, offspring were anesthetized by inhaled isoflurane (pre-oxygenation with oxygen alone for 4–5 breaths, starting off with 0.5% isoflurane and increasing the isoflurane percentage by 0.5% increments every few breaths to a maximum of 4%). Hearts were immediately excised and placed in ice-cold HEPES-buffered physiological saline solution (PSS; composition (in mmol/L): 10 HEPES, 5.5 glucose, 1.56 CaCl_2_, 4.7 KCl, 142 NaCl, 1.17 MgSO_4_, 1.18 KH_2_PO_4_, and pH 7.4) for assessment of ex vivo vascular function in the coronary arteries (see below). Plasma was collected using ethylenediaminetetraacetic acid (EDTA) anticoagulant, centrifuged, and stored at −80 °C for assessment of ET-1 levels. The main left coronary artery was isolated and immediately snapped frozen in optimal cutting temperature compound (Tissue-Tek^®^, Sakura Finetek, Torrance, CA, USA) for further molecular analysis. 

### 2.2. Coronary Artery Vascular Function by Wire Myography 

After collection of the heart, the left descending coronary artery (LADs; 150–250 μm) was isolated and cleaned from the myocardium in ice-cold PSS and divided into ~1–2 mm segments. Vascular function was assessed ex vivo using wire myography. The successful dissection and isolation of these vessels allowed us to obtain 1–3 segments of LAD per one heart. Subsequently, we used one to two offspring per one dam, in order to perform all the vascular function experiments (please see below). The data are presented based on the dam as the experimental unit; thus, duplicate (control) curves from 2 offspring from the same dam were averaged. The segments of LAD were mounted onto a wire myograph system (620M DMT, Copenhagen, Denmark) using 25 μm tungsten wires. Isomeric tension of the vessels was recorded using LabChart software (version 8.1.13; AD Instruments; Colorado Springs, CO, USA). All vessels were normalized through a series of stepwise increases in diameter to reach their optimal resting tension: 0.8 × IC100; 13.3 kPa (the internal circumference equivalent to a transmural pressure of 100 mmHg). After normalization, the vessels were exposed to the first wake up dose of the thromboxane A_2_ receptor agonist 9,11-Dideoxy-11α,9α-epoxymethanoprostaglandin F2α (U-46619; 1 μmol/L; Sigma-Aldrich, St. Louis, MO, United States) for 5 min. After washing with PSS thrice and 10 min rest, the vessels were exposed to a second wake up dose of U-46619, followed by a single dose of methylcholine (MCh; 3 μmol/L; Sigma-Aldrich), to confirm endothelial cell function. If LAD segments developed spontaneous sustained vasoconstriction during the experiment, the data obtained from those segments were excluded. After washing and 30 min of rest, vascular responses to MCh were assessed using a cumulative concentration response curve (CCRC; 0.001 μmol/L to 100 μmol/L MCh; doses were added in 2-min intervals) after pre-constriction with a pre-calculated EC_80_ concentration of U-46619 (0.2 μmol/L; the mean effective concentration that produces 80% of the maximal response). After washing with PSS thrice and 30 min of rest, vasoconstriction responses to ET-1 were assessed in LAD of 4-month-old and 9.5-month-old offspring using CCRCs to ET-1 (CCRC; 0.0001 μmol/L to 0.3 μmol/L; Sigma-Aldrich) in the presence or absence of ET_A_ (BQ-123; 10 μmol/L) or ET_B_ (BQ-788; 10 μmol/L) receptor antagonists [31]. As we observed impaired vasodilation to MCh at 4-months, which was maintained at 9.5 months of age, specific vasodilation pathways potentially involved in the impaired endothelium-dependent vasodilation were assessed in the 9.5-month-old offspring. CCRCs to MCh were performed in the absence or presence (30 min pre-incubation prior to U-46619 EC_80_ dose) of the following specific inhibitors: NO synthase (NOS) was inhibited with the pan NOS inhibitor N(G)-nitro-L-arginine methyl ester hydrochloride (L-NAME; 100 μmol/L; Sigma-Aldrich) [32], prostaglandin H synthase (PGHS) was inhibited with meclofenamate (10 μmol/L; Sigma-Aldrich), and EDH-mediated vasodilation was inhibited with a combination of apamin (0.5 μmol/L; Sigma-Aldrich) [33], which blocks small-conductance Ca^2+^-activated potassium channels (SK) and 1-(2-chlorophenyl)diphenylmethyl-1H-pyrazole (Tram-34; 0.4 μmol/L; Sigma-Aldrich) [33], an intermediate-conductance Ca^2+^-activated potassium channel (IK) inhibitor. Simultaneously, a CCRC to the NO donor sodium nitroprusside (SNP CCRC; 0.0001 μmol/L to 10 μmol/L, Sigma-Aldrich; doses were added in 2-min intervals) was conducted in one of the LAD segments to assess endothelium-independent relaxation. All data were presented as the maximum relaxation response to MCh (E_max_), the negative log of the mean effective concentration that produces 20% and 50% of the maximal response (pEC_20_ and pEC_50_, respectively), as a measure of vasodilator sensitivity, or as area under the curve (AUC).

### 2.3. Molecular Assessment of Plasma ET-1 Levels with ELISA

Plasma levels of ET-1 were assessed using a rat endothelin-1 ELISA kit (Boster Bio, Pleasanton, CA, USA, Cat# EK0952), according to the manufacturer’s instructions. Briefly, on the day of experiment, plasma samples were thawed on ice and vortexed. All reagents and working standards of the ELISA kit were prepared on the same day as the assay procedure. After adding all standards, controls, and samples in duplicate, plates were sealed and incubated for 90 min at 37°C. The plates were emptied, and each well was washed with washing solution 3 times. Next, biotinylated anti-rat Edn1 antibody was added in each well, sealed, and incubated for 60 min at 37°C. Afterwards, the plates were emptied and washed 3 times with washing buffer. Avidin-biotin-peroxidase complex was added in each well, and the plate was sealed and incubated for 30 min at 37°C. Plates were emptied, and each well was washed with washing solution 5 times. Color developing reagent was added to each well, and plates were sealed and incubated in the dark for 20 min at 37°C. Stop solution was added, and the absorbance of each well was measured under 450 nm wavelength within 30 min with multimode reader (BioTek Synergy HTX, Santa Clara, CA, USA). To determine the amount of ET-1 in the plasma sample, a standard curve was generated by plotting the average absorbance (450 nm) obtained for each of the eight standard ET-1 concentrations provided. The absorbance value was calculated for each standard and sample well, and all absorbance values (average of duplicates) were subtracted by the average of the zero-standard value (i.e., the blank). The levels of ET-1 in each plasma sample were calculated using the standard curve. 

### 2.4. Immunofluorescent Staining of the Main Left Coronary Artery for ET-1, ET_A_, ET_B_, eNOS, PGHS-1, and PGHS-2

Cryo-sections (9 µm) of coronary arteries of the 4- and 9.5-month-old offspring were prepared using a cryostat (SLEE medical GmbH, Nieder-Olm, Germany) for immunofluorescent detection of ET-1, endothelin A (ET_A_), and endothelin B (ET_B_) receptors, and for eNOS, PGHS-1, and PGHS-2 in coronary arteries of the 9.5-month-old offspring. Sections were fixed in ice cold methanol (−20°C for 10 min), then acetone (-20°C for 5 min), air-dried, and washed in phosphate buffer saline (PBS, pH: 7.5; 3 × 5 min). Afterwards, sections were subjected to an autofluorescence reduction treatment by incubating with NaBH_4_ in PBS (1 mg/mL) for 10 min at room temperature (RT) and washed with PBS (3 × 10 min). After incubation with blocking solution (PBS supplemented with 2% donkey serum and 1% bovine serum albumin (BSA) with 0.1% Triton X-100 in PBS) for 1 h at room temperature, sections were washed with PBS (3 × 10 min) and incubated overnight at 4°C in 1% BSA/PBS with the primary antibodies. The primary antibodies used were as follows: ET-1 (1:50; rabbit polyclonal anti-ET-1, Bioss Cat# bs-0954R, RRID:AB_10855124, USA), ET_A_ (1:300; rabbit polyclonal anti-ETA, Thermo Fisher Scientific, Waltham, MA; USA; Cat# PA3–065, RRID:AB_11153077), ET_B_ (1:50; rabbit polyclonal anti-ETB, Bioss Cat# bs-4198R, RRID:AB_11076171), eNOS (1:50; mouse monoclonal anti-eNOS (NOS Type III), BD Biosciences, Mississauga, ON, Canada; Cat# 610297, AB_397691), PGHS-1 (1:100; rabbit polyclonal anti-COX-1, Abcam, Waltham, MA, USA; Cat# ab53766, RRID:AB_879753), PGHS-2 (1:50; rabbit polyclonal anti-COX-2, Abcam Cat# ab52237, RRID:AB_869240), or CD31 (1:200; mouse monoclonal anti-PECAM-1, BD Pharmingen, Mississauga, ON, Canada; Cat# 550300, RRID:AB_393595). The next day, sections were washed with PBS (3 × 5 min) and incubated with secondary antibodies (all at 1:250; Thermo Fisher Scientific) with 4′,6′-diamidino-2-phenylindole (DAPI; 1:500 Thermo Fisher Scientific Cat# D3571, RRID:AB_2307445) in 1% BSA/PBS for 1 h at RT in the dark: donkey-anti-mouse Alexa Fluor™ (Thermo Fisher Scientific) 488 (Cat # A-21202, RRID: AB_141607) for eNOS, donkey-anti-mouse Alexa Fluor™ 546 (Cat # A10036, RRID:AB_2534012) for CD31, and donkey-anti-rabbit Alexa Fluor™ 488 (Cat # A-21206, RRID:AB_2535792) for ET-1, ET_A_, ET_B_, PGHS-1, and PGHS-2. After washing in PBS (3 × 10 min), mounting medium containing DAPI was added (Vector Laboratories, Burlingame, CA, USA), and slides were stored in the dark and left to dry overnight. Immunofluorescent images were obtained the next day using a confocal Zeiss LSM 700 microscope with Zen Black software (version 8.1.6.484; Zeiss, Toronto, ON, Canada). Gains for AF546, AF488, and DAPI were set using the blank sections (i.e., incubated with secondary antibody only).

### 2.5. Image Analysis of Immunofluorescence Staining

Fluorescent images of ET-1, ET_A_, ET_B_, eNOS, PGHS-1, and PGHS-2 staining in the coronary arteries were analyzed using FIJI ImageJ software (version 1.53n; Wayne Rasband NIH, Bethesda, MD, USA). The total vessel area was selected for ET-1, ET_A_, eNOS, PGHS-1, and PGHS-2, the vessel area outside of the endothelium (the area negative for CD31) was selected for ET_B_ (ET_B_ that are located on VSMCs mediate vasoconstriction [34]), and the mean fluorescent intensity was measured. 

### 2.6. Statistical Analysis

Data from the dose response curves were fitted to the Hill equation, and pEC_50_ and pEC_20_ values were calculated. The pEC_50_ and pEC_20_ values are defined as the negative logarithm of the EC_50_ and EC_20_ (the concentration of agonist that provokes a response 50% and 20% of maximum response, respectively) and represent receptor sensitivity to the agonist. For methylcholine (in the presence or absence of L-NAME) and ET-1 (in the presence or absence of BQ123 or BQ788) dose response curves, the area under the curve (AUC) was calculated as a cumulative measurement of an inhibitor effect. Data were analyzed by student *t*-test or two-way ANOVA with Sidak’s post-hoc test (GraphPad Prism, version 9.1.2; San Diego, CA, USA; https://www.graphpad.com/scientific-software/prism/). Data were presented as means  ± standard error of the mean (SEM); *p <* 0.05 was considered statistically significant. 

## 3. Results

### 3.1. Coronary Artery Endothelium-Dependent and Endothelium-Independent Vasodilation Responses

#### 3.1.1. Endothelium-Dependent Vasodilation Was Impaired in Prenatally Hypoxic Male and Female 4-Month-Old Offspring

**Figure 1:**
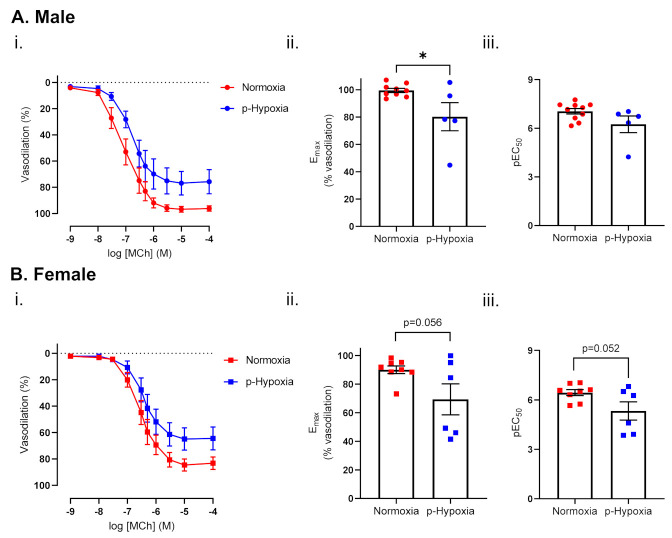
Endothelium-dependent vasodilation in 4-month-old male (**A**) and female (**B**) offspring exposed to prenatal hypoxia. (**i**) Endothelium-dependent vasodilation responses to increasing doses of methylcholine (MCh) in left descending coronary arteries of Normoxia (red) and p-Hypoxia (blue) male (circles) and female (squares) 4-month-old offspring. (**ii**) Data were summarized as maximal vasodilation to MCh (E_max_) and (**iii**) the sensitivity to MCh (the negative logarithm of the EC_50_, pEC_50_), *n* = 5–10 dams/group. Data are presented as mean ± SEM; analyzed by t-test; * *p <* 0.05.

MCh-induced vasodilation responses were decreased in p-hypoxia males, while the sensitivity to MCh was comparable between normoxia and p-hypoxia groups. In females, MCh-induced vasodilation responses and sensitivity to MCh tended to decrease in the p-hypoxia group, compared to normoxia control (Figure 1A,B). In contrast, no changes in the vascular responses to the NO donor (SNP) were observed in the coronary arteries of 4-month-old male (E_max_: normoxia: 98.95 ± 1.11 vs. p-hypoxia: 94.11 ± 1.51, *n* = 6–8 dams/group) or female offspring (E_max_: normoxia: 98.56 ± 0.79 vs. p-hypoxia: 96.83 ± 1.75, *n* = 6–8 dams/group).

#### 3.1.2. Impaired Endothelium-Dependent Vasodilation in Prenatally Hypoxic Male and Female 9.5-Month-Old Offspring

**Figure 2:**
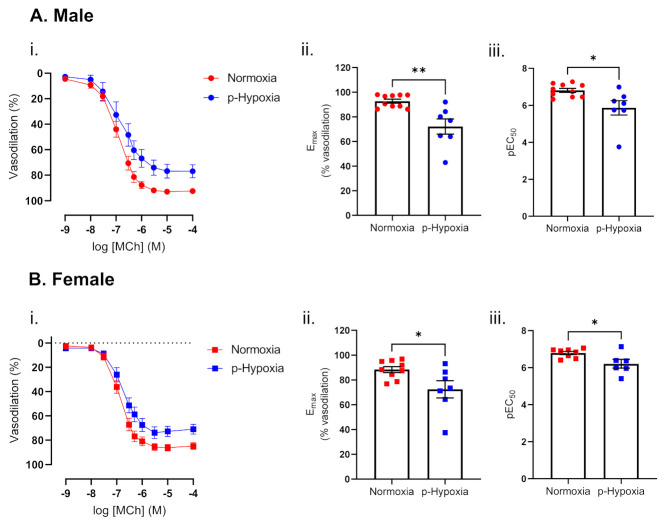
Endothelium-dependent vasodilation in 9.5-month-old male (**A**) and female (**B**) offspring exposed to prenatal hypoxia. (**i**) Endothelium-dependent vasodilation responses to increasing doses of methylcholine (MCh) in left descending coronary arteries of Normoxia (red) and p-Hypoxia (blue) male (circles) and female (squares) 9.5-month-old offspring. (**ii**) Data were summarized as maximal vasodilation to MCh (E_max_) and (**iii**) the sensitivity to MCh (the negative logarithm of the EC_50_, pEC_50_), *n* = 7–10 dams/group. Data are presented as mean ± SEM; analyzed by *t*-test; * *p <* 0.05, ** *p <* 0.01.

With an advancement in age, at 9.5 months of age, maximal vasodilation responses to MCh and the sensitivity to MCh were decreased in the coronary arteries of p-hypoxia males and females (Figure 2A,B). SNP-mediated vasodilation responses were similar between normoxia and p-hypoxia males (E_max_ normoxia: 100.02 ± 0.0.37 vs. p-hypoxia: 98.47 ± 1.37, *n* = 6–8 dams/group) and females (E_max_ normoxia: 98.95 ± 0.66 vs. p-hypoxia: 97.73 ± 2.65, *n* = 6–8 dams/group).

### 3.2. Mechanisms of Endothelium-Dependent Vasodilation in Male and Female 9.5-Moth-Old Offspring

Because an impairment in MCh-induced vasodilation was maintained at 9.5 months of age in both male and female offspring, specific vasodilation pathways potentially involved in the impaired endothelium-dependent vasodilation were assessed in the 9.5-month-old offspring.

#### 3.2.1. The Nitric Oxide Synthase (NOS) Pathway Is a Major Contributor to Coronary Artery Endothelium-Dependent Vasodilation in Male and Female Offspring

**Figure 3:**
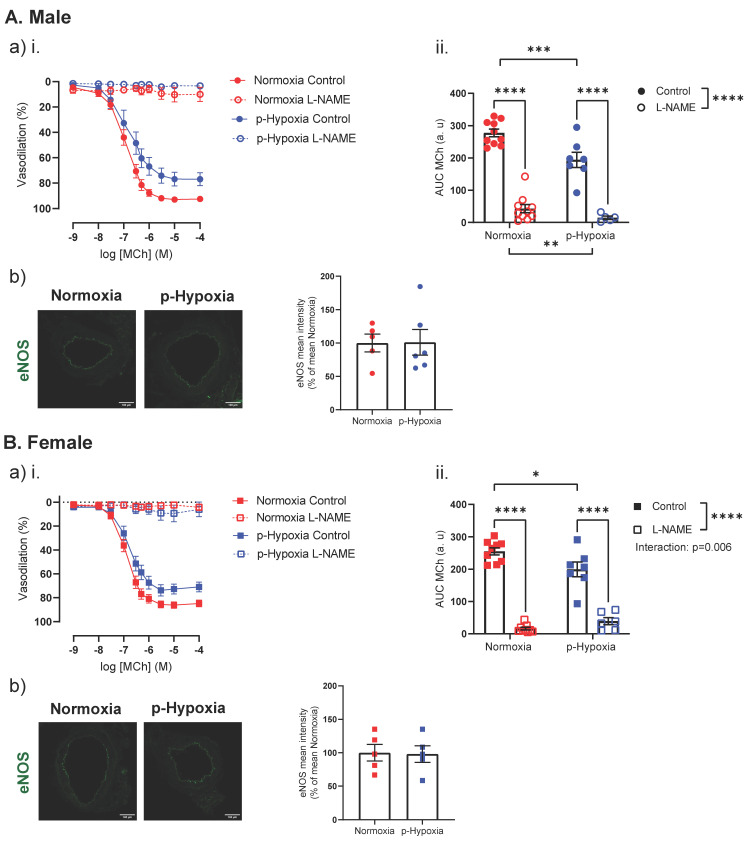
Contribution of nitric oxide to coronary artery endothelium-dependent vasodilation in 9.5-month-old male and female offspring. (**i**) Endothelium-dependent vasodilation responses to increasing doses of methylcholine (MCh) in the presence or absence of L-NAME (NOS inhibitor N(G)-nitro-L-arginine methyl ester hydrochloride) in left descending coronary arteries of Normoxia (red) and p-Hypoxia (blue) male (**A**; circles) and female (**B**; squares) 9.5-month-old offspring. (**ii**) Data are summarized as area under the curve (AUC) in the absence or presence of L-NAME, *n* = 5–10 dams/group. (**iii**) Representative confocal images of immunofluorescence staining of eNOS (green) and quantitative analysis of eNOS expression in coronary arteries of Normoxia (red symbol) and p-Hypoxia (blue symbol) male (**A**; circles) and female (**B**; squares) 9.5-month-old offspring; *n* = 5–6 dams/group. Data are presented as mean ± SEM; analyzed by two-way ANOVA with Sidak’s multiple comparison post-hoc test or *t*-test, * *p <* 0.05, *** *p <* 0.001, **** *p <* 0.0001.

Pre-incubation with L-NAME prevented MCh-induced vasorelaxation in normoxia and p-hypoxia male offspring (Figure 3A). Because eNOS is a predominant NOS isoform in the vasculature and is responsible for most of the NO produced, we assessed the eNOS protein expression in the coronary artery tissue of the offspring. The eNOS expression was similar between normoxia and p-hypoxia male offspring (Figure 3A). Similar to the male offspring, in the coronary arteries of female offspring, pre-incubation with L-NAME prevented MCh-induced vasorelaxation in the normoxia and p-hypoxia groups (*p <* 0.0001; Figure 3B). eNOS levels were similar between the normoxia and p-hypoxia groups (Figure 3B).

#### 3.2.2. Enhanced Contribution of the PGHS Pathway to Coronary Artery Endothelium-Dependent Vasodilation in Prenatally Hypoxic Offspring

**Figure 4:**
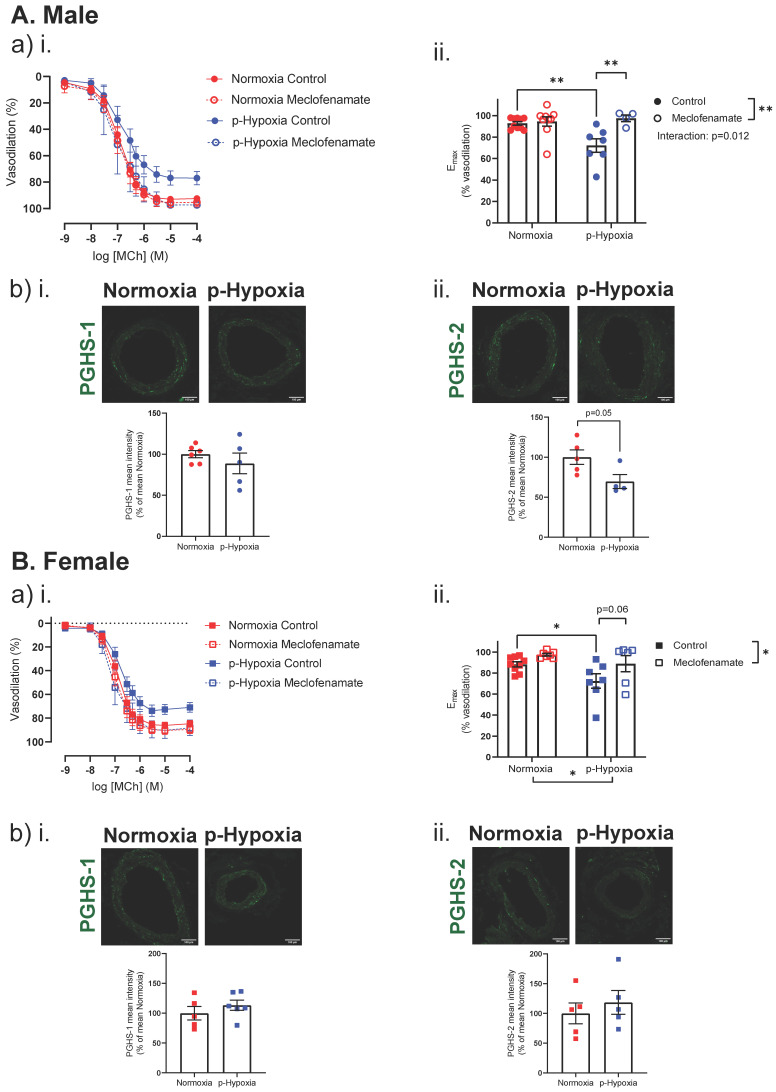
Contribution of the prostaglandin-H synthase (PGHS) pathway to endothelium-dependent vasodilation in 9.5-month-old male and female offspring. (**a**). (**i**) Endothelium-dependent vasodilation responses to increasing doses of methylcholine (MCh) in the presence or absence of meclofenamate (a PGHS inhibitor) in left descending coronary arteries of Normoxia (red) and p-Hypoxia (blue) male (**A**; circles) and female (**B**; squares) 9.5-month-old offspring. (**ii**) Data are summarized as maximum vasodilation response (E_max_) in the absence or presence of meclofenamate; *n* = 4–10 dams/group. (**b**). (**i**,**ii**) Representative confocal images of PGHS-1 (green) and PGHS-2 (green) expression, and quantitative analysis of immunofluorescence staining for PGHS-1 and PGHS-2 in coronary arteries of Normoxia (red) and p-Hypoxia (blue) male (circles) and female (squares) offspring; *n* = 4–6 dams/group. Data are presented as mean ± SEM; analyzed by *t*-test or two-way ANOVA with Sidak’s multiple comparison post-hoc test, * *p <* 0.05, ** *p <* 0.01.

In the male offspring, pre-incubation with meclofenamate did not affect MCh-induced vasorelaxation in the normoxia group, while meclofenamate increased the max MCh-induced vasodilation in the p-hypoxia offspring. Since PGHS-1 and PGHS-2 are key enzymes involved in the conversion of arachidonic acid to prostaglandins (PGs) and other eicosanoids, coronary artery tissue expression of PGHS-1 and PGHS-2 was assessed. Coronary artery PGHS-1 expression was similar between the normoxia and p-hypoxia male offspring, while the expression of PGHS-2 was lower (*p =* 0.050) in the p-hypoxia group, compared to the normoxia male offspring (Figure 4A). Similar to the male offspring, in females, pre-incubation with meclofenamate tended to increase (*p =* 0.06) MCh-induced vasorelaxation in only the p-hypoxia offspring (Figure 4B). Coronary artery expressions of PGHS-1 and PGHS-2 were not different between the normoxia and p-hypoxia groups (Figure 4B).

#### 3.2.3. The Contribution of Endothelium-Derived Hyperpolarization (EDH) to Endothelium-Dependent Vasodilation Is Enhanced in Coronary Arteries of Prenatally Hypoxia Male and Female Offspring

**Figure 5:**
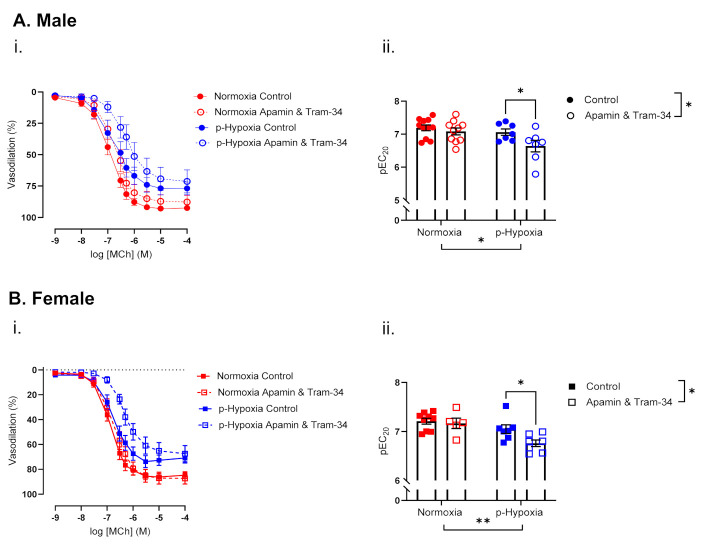
Contribution of endothelium-derived hyperpolarization to endothelium-dependent vasodilation in 9.5-month-old male and female offspring. (**i**) Endothelium-dependent vasodilation responses to increasing doses of methylcholine (MCh) in the presence or absence of apamin and Tram-34 (small- and intermediate-conductance Ca^2+^-activated potassium channels inhibitors) in left descending coronary arteries of Normoxia (red) and p-Hypoxia (blue) male (**A**; circles) and female (**B**; squares) 9.5-month-old offspring. (**ii**) Data are summarized as the sensitivity to MCh (the negative logarithm of the EC_20_, pEC_20_); *n* = 5–9 dams/group. Discontinuous line for Figure 5Aii and Figure 5Bii is used to better visualize the data. Data are presented as mean ± SEM; analyzed by two-way ANOVA with Sidak’s multiple comparison post-hoc test, * *p <* 0.05, ** *p <* 0.01.

In the males, pre-incubation with apamin and Tram-34 decreased coronary artery sensitivity to MCh in the p-hypoxia group only (Figure 5A). In females, apamin and Tram-34 did not alter coronary artery sensitivity to MCh in normoxia group, while it decreased coronary artery sensitivity to MCh in the p-hypoxia offspring (Figure 5B).

### 3.3. Coronary Artery Responses to ET-1 and the Contribution of ET_A_ and ET_B_

#### 3.3.1. Increased Contribution of ET_B_ Receptors to ET-1 Mediated Vasoconstriction in 4-Month-Old Female Offspring

**Figure 6:**
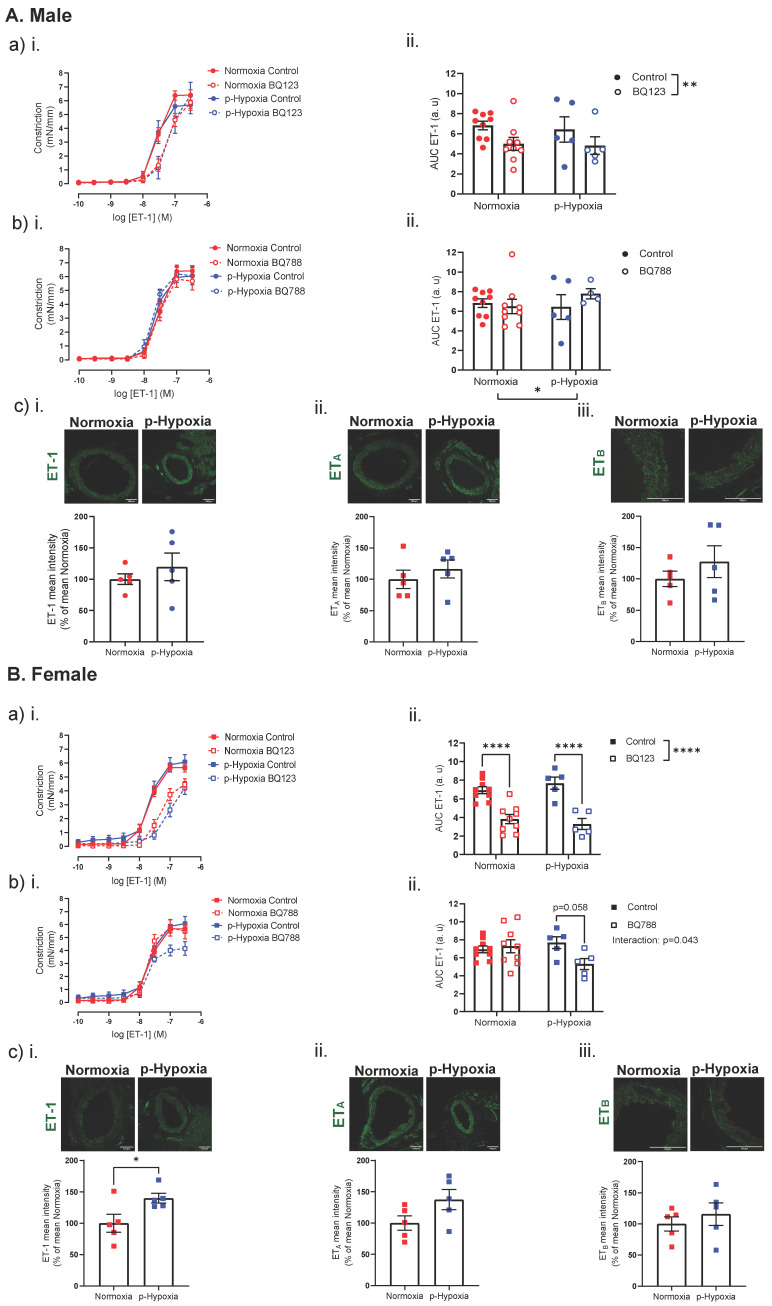
ET-1-mediated vasoconstriction, contribution of ET_B_ to ET-1-mediated response and coronary tissue levels of ET-1 and ET_B_ in 4-month-old male and female offspring. (**i**) ET-1-mediated vasoconstriction in the presence or absence of (**a**) BQ123 (a competitive antagonist of ET_A_) or (**b**) BQ788 (a selective ET_B_ receptors antagonist) in left descending coronary arteries of Normoxia (red) and p-Hypoxia (blue) male (**A**; circles) and female (**B**; squares) 4-month-old offspring. (**ii**) Data are summarized as area under the curve (AUC); *n* = 4–9 dams/group. (**c**) Representative confocal images of (**i**) ET-1 (green), (**ii**) ET_A_ (green) and (**iii**) ET_B_ (green) co-stained with CD31 (endothelial cell marker; red) and quantitative analysis of immunofluorescence staining in Normoxia (red symbol) and p-Hypoxia (blue symbol) male (circles) and female (squares) offspring; *n* = 4–6 dams/group. Data are presented as mean ± SEM; analyzed by *t*-test or two-way ANOVA with Sidak’s multiple comparison post-hoc test, ** *p <* 0.01, **** *p <* 0.0001.

In 4-months-old male offspring, ET-1-mediated coronary artery responses and ET-1 expression were similar between the normoxia and p-hypoxia groups (Figure 6A). Moreover, ET-1 plasma levels were comparable between normoxia and p-hypoxia males (normoxia: 1.40 ± 0.19 pg/mL vs. p-hypoxia: 1.13 ± 0.36 pg/mL, *n* = 6–7 dams/group).

BQ123 (a competitive antagonist of ET_A_) decreased the vasoconstriction responses to ET-1 to a similar extent in both the normoxia and p-hypoxia males (Figure 6A). Coronary artery ET_A_ expression was similar between normoxia and p-hypoxia male offspring (Figure 6A). Pre-incubation with BQ788 (a non-competitive antagonist of ET_B_ receptors) did not alter ET-1-mediated vasoconstriction in the normoxia group and p-hypoxia group (Figure 6A). ET_B_ expression was not different between normoxia and p-hypoxia male offspring (Figure 6A).

In 4-month-old females, ET-1-mediated coronary artery constriction responses were similar between the normoxia and p-hypoxia groups (Figure 6B). The ET-1 expression was increased in the p-hypoxia females, compared to normoxia group (Figure 6B), while the plasma ET-1 levels were similar between the groups (normoxia: 2.55 ± 0.35 pg/mL vs. p-hypoxia: 1.60 ± 0.48 pg/mL, *n* = 7–8 dams/group). BQ123 decreased ET-1-mediated vasoconstriction in both the normoxia and p-hypoxia offspring, while no significant differences in ET_A_ expression were observed between the groups (Figure 6B). BQ788 did not alter ET-1-mediated coronary artery responses in the normoxia offspring, while ET-1-mediated vasoconstriction tended to be decreased (*p* = 0.058) by BQ788 in the p-hypoxia offspring (Figure 6B). However, ET_B_ expression was similar between the normoxia and p-hypoxia groups (Figure 6B).

#### 3.3.2. An Impaired ET-1 Mediated Vasoconstriction in 9.5-Month-Old Female Offspring

**Figure 7:**
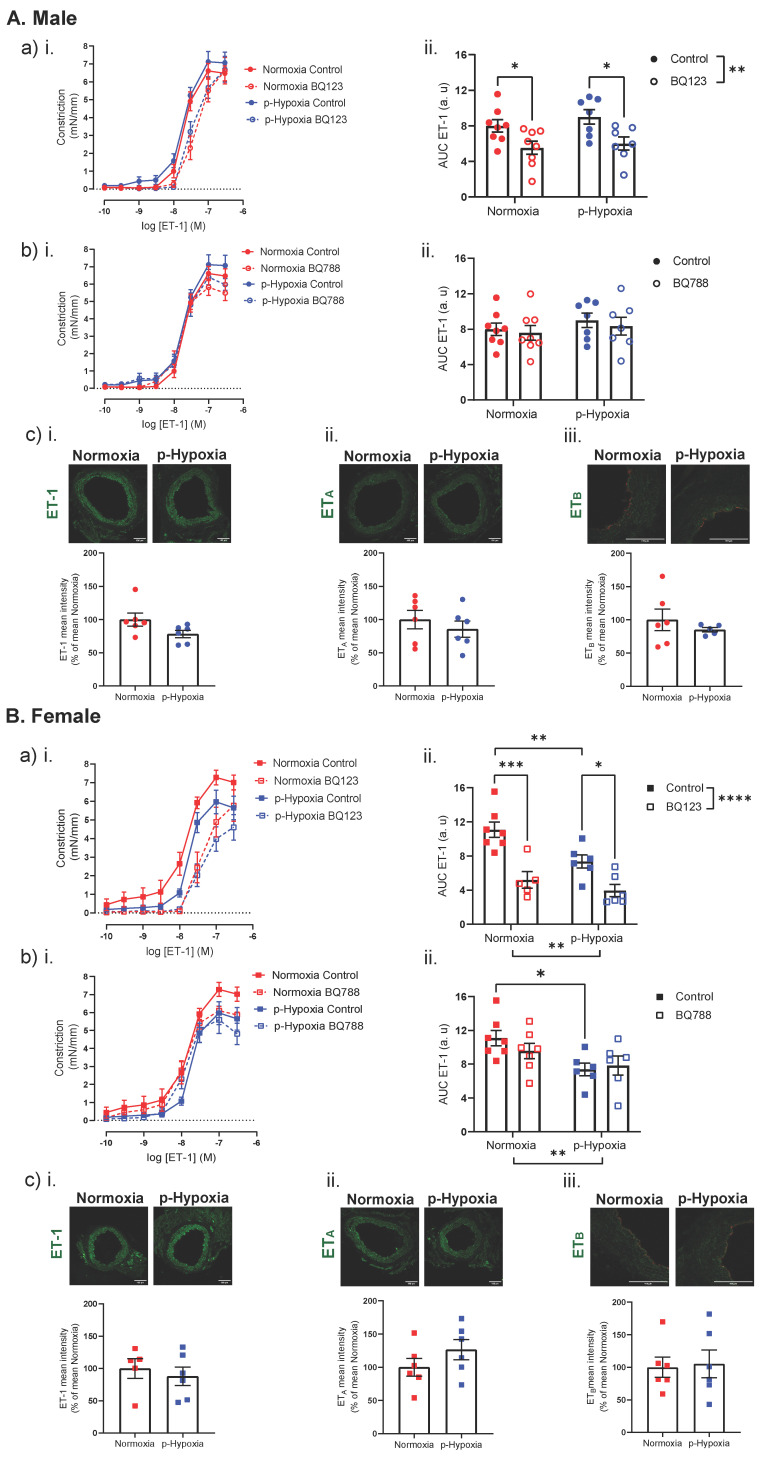
ET-1-mediated vasoconstriction, contribution of ET_B_ to ET-1-mediated response and coronary tissue levels of ET-1 and ET_B_ in 9.5-month-old male and female offspring. ET-1-mediated vasoconstriction in the presence or absence of (**a**) BQ123 (a competitive antagonist of ET_A_) or (**b**) BQ788 (a selective ET_B_ receptors antagonist) in left descending coronary arteries of Normoxia (red) and p-Hypoxia (blue) male (**A**; circles) and female (**B**; squares) 9.5-month-old offspring. (**ii**) Data are summarized as area under the curve (AUC); *n* = 5–8 dams/group. (**c**) Representative confocal images of (**i**) ET-1 (green), (**ii**) ET_A_ (green), and (**iii**) ET_B_ (green) co-stained with CD31 (endothelial cell marker; red) and quantitative analysis of immunofluorescence staining in Normoxia (red symbol) and p-Hypoxia (blue symbol) male (circles) and female (squares) offspring; *n* = 5–6 dams/group. Data are presented as mean ± SEM; analyzed by *t*-test or by two-way ANOVA with Sidak’s multiple comparison post-hoc test, * *p <* 0.05, ** *p <* 0.01, *** *p <* 0.001, **** *p <* 0.0001.

In 9.5-months-old male offspring, ET-1 mediated coronary artery responses and coronary tissue expression of ET-1 were similar between the normoxia and p-hypoxia groups (Figure 7A). Plasma levels of ET-1 were similar between normoxia and p-hypoxia male offspring (normoxia: 4.65 ± 0.86 pg/mL vs. p-hypoxia: 4.11 ± 0.67 pg/mL, *n* = 7 dams/group). BQ123 (a competitive antagonist of ET_A_) decreased, to a similar extent, the vasoconstriction responses to ET-1 in both the normoxia and p-hypoxia males (Figure 7A). ET_A_ expression was similar between normoxia and p-hypoxia males (Figure 7A). Pre-incubation with BQ788 (a selective ET_B_ antagonist) did not alter ET-1-mediated coronary artery responsiveness in the normoxia and p-hypoxia males, and coronary expression of ET_B_ was similar between normoxia and p-hypoxia groups (Figure 7A).

In female offspring, ET-1 mediated responses were reduced in the p-hypoxia group, compared to the normoxia controls (Figure 7B). The ET-1 tissue levels were similar between normoxia and p-hypoxia groups (Figure 7B), while the plasma levels tended to be lower in the p-hypoxia groups, compared to normoxia (normoxia: 10.47 ± 0.82 pg/mL vs. p-hypoxia: 7.67 ± 1.02 pg/mL; *p* = 0.065, *n* = 6–8 dams/group). Pre-incubation with BQ123 (a competitive antagonist of ET_A_ receptors) decreased the vasoconstriction responses to ET-1 in the normoxia and p-hypoxia females, while the coronary tissue levels of ET_A_ were similar between the normoxia and p-hypoxia groups (Figure 7B). Pre-incubation with BQ788 (a selective ET_B_ antagonist) did not alter the ET-1-mediated coronary artery responsiveness in the normoxia and p-hypoxia females, and coronary expression of ET_B_ was similar between normoxia and p-hypoxia groups (Figure 7B).

## 4. Discussion

In the current study, we demonstrated that hypoxia, experienced during prenatal life, is associated with changes in the vasoconstrictor and vasodilator capacity of coronary arteries in adult male and female offspring. We showed that prenatal hypoxia impairs endothelium-dependent vasodilation in both males and females at 4- and 9.5-month of age. Nitric oxide was the predominant vasodilatory pathway in coronary circulation. However, in both males and females exposed to prenatal hypoxia, the impaired endothelium-dependent vasodilation appeared to be mediated via enhanced prostaglandin H-synthase-dependent vasoconstriction. Moreover, a higher contribution of endothelium-dependent hyperpolarization to coronary artery vasodilation was observed in prenatally hypoxic males and females. In 4-month-old offspring, prenatal hypoxia did not affect ET-1-mediated responsiveness, but tended to increase the contribution of ET_B_ to ET-1 vasoconstriction in female offspring only. With advancement in age, vasoconstriction responses of the coronary arteries to ET-1 were reduced by prenatal hypoxia in only the female offspring, without effects of prenatal hypoxia on ET_B_-mediated responses in either sex.

### 4.1. The Effect of Prenatal Hypoxia on Coronary Artery Endothelium-Dependent and Endothelium-Independent Vasodilation in Adult Male and Female Offspring

To the best of our knowledge, we showed, for the first time, that prenatal exposure to hypoxia impairs endothelium-dependent vasodilation in coronary arteries of male and female offspring at 4 and 9.5 months of age. The coronary endothelium regulates vascular tone by releasing vasoconstricting and vasodilating factors, and an impairment in endothelium-dependent vasodilation has been shown to contribute to the development of various CV pathophysiological states [35], such as hypertension [36], atherosclerotic vascular disease [37], and congestive heart failure [38]. We, and others, have previously reported an impaired cardiac tolerance to ischemia/reperfusion (I/R) injury in the offspring exposed to prenatal hypoxia [39,40,41,42]. As impaired vasodilation of the coronary microcirculation has been associated with defects in myocardial perfusion (suggestive of myocardial ischemia) [43], it may be speculated that the impaired functional properties of the coronary arteries, observed in the current study, are a significant contributor factor to the development of cardiac dysfunction of adult offspring.

Further assessment of the potential mechanisms of the impaired endothelium-dependent vasodilation in the 9.5-month-old offspring revealed that, in the LAD, endothelium-dependent vasodilation is predominantly mediated via NO, which has been previously reported in large epicardial coronary arteries [44,45]. NO is produced in the tissue by conversion of L-arginine to L-citrulline by NOS [46]. eNOS is critical for normal vascular homeostasis and is responsible for generating endothelial-derived NO [47]. Previous studies reported that coronary artery endothelial dysfunction may be associated with an increase [48], as well as decrease [49,50], in eNOS expression. In the current study, however, we did not observe any changes in eNOS expression in either males or females due to prenatal hypoxia.

Interestingly, in 9.5-month-old adult male and female offspring, we observed that inhibition of PGHS improved the impairment in MCh-induced vasorelaxation induced by prenatal hypoxia. PGHS is an enzyme involved in the production of PGs, which include vasodilator (PGI_2_; prostacyclin), as well as the vasoconstrictor, compounds (prostaglandin H_2_, prostaglandin F2α and TXA_2_). PGs are critical modulators of vascular tone in both physiological and pathophysiological conditions (reviewed by [51,52]). Because the inhibition of PGHS improved MCh-induced vasorelaxation in prenatally hypoxia males and females, PGHS expression was assessed in the coronary artery tissue. Prenatal hypoxia did not alter PGHS-1 and PGHS-2 expression in females and PGHS-1 expression in males, which suggests that not the levels but the activity of the PGHS pathway involved in the production and action of constrictive PGs or TXA_2_ may be enhanced by prenatal hypoxia, thereby contributing to an impaired vasodilation. In contrast to the females, the expression of PGHS-2 was decreased in the prenatally hypoxic male offspring. Although PGHS-2 expression and activity is enhanced in various pathophysiological conditions, such as preeclampsia, hypertension, and ageing (reviewed in [51]), previous research demonstrated an essential role of PGHS-2 in postnatal CV maturation (the transition of the cardiopulmonary circulation at birth) [53] and in the maintenance of normal renal architecture (progression of the renal dysplasia seen in COX (PGHS)-2–deficient mice) [54], and PGHS-2 downregulation has been shown to contribute to the development of kidney pathologies, due to intrauterine growth restriction [55]. Thus, it may be suggested that a lower expression of PGHS-2 in the coronary arteries of the adult male offspring may be an indication of an early-life adaptation of the coronary circulation to adverse intrauterine environment during pregnancies complicated with prenatal hypoxia.

We observed that, in normoxic and prenatally hypoxic male and female offspring, endothelial hyperpolarization through SK_Ca_ and IK_Ca_ channels appears to contribute to endothelium-dependent vasodilation. Both the SK_Ca_ and IK_Ca_ channels play an important channel-specific role in the endothelium-dependent vasodilation (reviewed by [56,57]). For instance, SK_Ca_ are mainly expressed in caveolae and are important for the activation of NOS, while IK_Ca_ can be mainly found within myoendothelial gap junction-associated endothelial cell projections [58]. SK_Ca_ and IK_Ca_ channel activation generates an endothelium-dependent hyperpolarization that is conducted along the endothelium and into the smooth muscle cell layer, leading to smooth muscle cell hyperpolarization and subsequent inhibition of vascular tone [59]. An impaired function of SK_Ca_ and IK_Ca_ channels has been observed in various CV pathologies [60,61]. Moreover, it has been reported that hypoxia, per se, leads to a reduction in the expression level of SK_Ca_ and IK_Ca_, as well as a reduction in endothelial K^+^ currents via SK_Ca_ and IK_Ca_ channels in porcine coronary arteries (in either sex) [62]. Thus, it may be suggested that the EDH pathway is maintained in prenatally hypoxic males and females as an additional mechanism to compensate for an impaired relaxation capacity of coronary arteries, thus maintaining coronary artery vasorelaxation in males and females.

### 4.2. The Effect of Prenatal Hypoxia on Coronary Artery ET-1 System in Male and Female Offspring

At 4 months of age, ET-1 tissue levels were increased in coronary arteries from prenatal hypoxia females only, while ET-1-mediated vasoconstriction and plasma levels of ET-1 were not affected by prenatal hypoxia in either sex. Previous research in an adult rat model of chronic intermittent hypoxia reported an enhanced ET-1 expression in coronary vessels that was accompanied with an enhanced ET-1-mediated response [63]. Thus, it can be suggested that prenatal hypoxia may have an adaptive response in that, although there was an increase in ET-1 expression, this was not accompanied by an increased ET-1-mediated coronary artery responsiveness.

The biological effects of ET-1 are achieved via activation of the ET_A_ and ET_B_ [34]. In the vasculature, ET_B_ are located on the endothelium and VSMCs. The upregulation of ET_B_ on VSMCs is often observed in atherosclerosis [64] and ischemic heart disease [65], as their activation potentiates ET-1-mediated vasoconstriction. On the VSMCs, ET_B_ activation induces the activation of phospholipase C-β, which results in the increase in inositol 1,4,5-trisphosphate and diacylglycerol (DAG). DAG activates PKC, which phosphorylates the actin-binding protein calponin or leads to phosphorylation of caldesmon, thereby increasing the myofilament force sensitivity to Ca^2+^, resulting in constriction [34]. Additionally, previous research has demonstrated a sex-specific function of ET_B_ [66]. Thus, in men, the activation of ET_B_ mediates tonic vasoconstriction in the blood vessels of the skin, while in females, it results in tonic vasodilation in the same type of blood vessels [66]. We demonstrated that, in 4-month-old prenatally hypoxic male offspring, the inhibition of ET_B_ receptors did not alter ET-1-mediated vasoconstriction, while in prenatal hypoxic female offspring, ET_B_ inhibition tended to decrease ET-1-mediated vasoconstriction. In a rat model of prenatal hypoxia, Chen et al. previously showed an attenuated constriction of coronary arteries of male (5-month-old; females were not assessed) offspring, which was associated with a decreased PKCβ^Ser660^ phosphorylation [24]. However, as our functional results were not associated with changes in ET_B_ expression, and it may be speculated that it is the downstream signaling of ET_B_ receptors that was impacted by prenatal hypoxia in female offspring, which resulted in sex-specific changes in an ET_B_-dependent functional response.

In 9.5-month-old offspring, prenatal hypoxia decreased ET-mediated coronary artery responsiveness in female offspring and tended to decrease the plasma levels of ET-1 (likely due to reduced ET-1 release or an enhanced ET-1 clearance from the circulation). While the long-term effect of prenatal hypoxia on the coronary artery ET-1 system is currently unknown, a decreased ET-1-mediated vasoconstriction in coronary arteries has been previously reported in obese rats, and this was associated with the uncoupling of [Ca^2+^]_i_ signaling, while ET-1, ET_A_, and ET_B_ expressions were not changed [67]. Moreover, a reduction in ET-1-induced coronary artery contraction has been observed in deoxycorticosterone acetate (DOCA)-salt hypertensive rats, which was associated with the uncoupling of ET-1-receptors and impaired [Ca^2+^]_i_ signaling [68]. Thus, it may be that the reduction in ET-1-mediated vasoconstriction in 9.5-month-old females is attributed to impaired [Ca^2+^]_i_ signaling; however, further studies are warranted.

## 5. Conclusions

In the current study, we showed that prenatal hypoxia has long-term and sex-specific effects on the mechanisms of coronary artery vasodilation and vasoconstriction. As coronary artery function is essential in maintaining cardiac performance, coronary artery dysfunction may contribute to the development of cardiac dysfunction and an impaired cardiac tolerance to I/R insult in adult offspring that has been previously reported [39,41,42]. Understanding the mechanistic pathways involved in the programming of CV disease allows for the development of future prenatal and postnatal therapeutic interventions.

## Data Availability

The data presented in this study are available on request from the corresponding author. The data are not publicly available, due to privacy.
